# Body and Brain

**Published:** 1858-10

**Authors:** 


### BODY AND BRAIN.

Motion is the exercise of the body. Thought is the exercise of the brain. Motion at length exhausts the body. Thought at length exhausts the brain. Cessation of motion allows the body to be invigorated. Cessation of thought reinvigorates the brain. The body must have rest. The brain must have sleep. When the body cannot rest, as in convulsive diseases, it dies. When the brain cannot rest, when a man cannot sleep, every hour is a step nearer to the mad-house. Some men work themselves to death. Some men think themselves to death. Too little rest for the body, too little sleep for the brain, are false economies of time; and multitudes, unwittingly, bring on wasting and fatal diseases by practicing these economies. Omnipotence "rested," and commanded man to do the same. Sleep a plenty, rest a plenty—these are the foundations of all great, safe, and efficient activities of body or brain.

